# Polymer-Functionalized Nanocatalysts: Engineering Interfaces and Microenvironments for Enhanced Catalysis

**DOI:** 10.3390/polym18040465

**Published:** 2026-02-12

**Authors:** Zhiyi Sun, Shuo Wang, Xuemin Hu

**Affiliations:** College of Textile and Garments, Textile and Garment Technology Innovation Center, Hebei University of Science and Technology, Shijiazhuang 050018, China

**Keywords:** polymer functionalization, nanocatalysts, interfacial engineering, microenvironment regulation, catalytic performance

## Abstract

Polymer functionalization is rapidly emerging as a transformative strategy for enhancing nanocatalysts by reprogramming the catalytic interface, rather than simply modifying the active phase. This approach leverages the unique tunability of polymers through their chemistry, thickness, permeability, charge density, and ionic/electronic conductivity to stabilize nanophases, regulate local microenvironments, and manage mass transport. These properties significantly improve catalytic activity, selectivity, and long-term durability. This review provides an in-depth examination of key construction strategies for polymer-functionalized nanocatalysts, categorizing them into six primary platforms: neutral functional polymers, ionomers/polyelectrolytes, conductive polymers, crosslinked networks/hydrogels, hybrid polymers, and framework polymers. Additionally, we explore recent advances in electrocatalysis, photocatalysis, and thermocatalysis, addressing challenges such as the trade-off between protection and accessibility, polymer stability under extreme conditions, and the need for standardized reporting of polymer descriptors. By framing polymers as programmable interfacial materials, this review highlights their potential to unlock significant improvements in catalytic performance across various catalytic systems.

## 1. Introduction

Polymers occupy a distinctive niche in materials chemistry because they unite structural versatility with chemical programmability [1,2,3,4,5]. Compared with small-molecule ligands or rigid inorganic coatings, polymer chains can present dense functional groups, adapt to curved nanosurfaces, and form continuous interfacial layers with tunable thickness, permeability, and mechanical compliance [6,7,8,9]. Their chemical space is similarly broad: polarity, charge density, hydrophobicity, hydrogen-bonding propensity, and coordination strength can be systematically tuned through monomer choice, copolymer architecture, and post-functionalization [10,11,12,13,14,15]. From a synthesis perspective, polymers are compatible with scalable deposition routes, including solution casting, dip-coating, layer-by-layer assembly, and in situ polymerization, and they integrate readily into complex catalyst architectures such as porous electrodes and membrane assemblies [16,17,18,19]. Collectively, these attributes position polymers not as passive binders but as programmable interfacial materials that control how molecules, ions, and electrons access and react at catalytic sites.

This advantage of polymers is particularly prominent for nanocatalysts [20,21,22,23,24,25,26]. Their high surface area and abundant low-coordination sites can deliver exceptional intrinsic activity, yet they also create persistent practical bottlenecks [27,28,29,30]. Under thermal or electrochemical operation, nanoparticles may aggregate or sinter, undergo surface reconstruction, leach or dissolve, and become fouled by strongly bound intermediates [31,32,33,34,35]. Moreover, catalytic performance is often governed not only by the active site itself but also by the local reaction microenvironment [36,37,38,39]. Reactant enrichment or depletion, local pH shifts, ionic strength, solvent organization, and mass-transport limitations can each reshape apparent activity and selectivity [40,41,42,43]. Conventional strategies typically tackle these issues one at a time—for example, by alloying to tune adsorption energetics, using oxide supports to anchor particles, or applying protective shells to enhance durability [44,45,46,47,48,49]. However, these approaches rarely optimize activity, selectivity, and stability simultaneously, and they often lack an explicit handle to engineer the microenvironment immediately adjacent to the active surface.

In this context, polymer functionalization has emerged as a powerful and increasingly general strategy in catalyst design [50,51,52,53,54]. In practice, polymers are deployed as ligands, brushes, shells, networks, or framework-like coatings to engineer the catalytic interface. Conceptually, polymers couple multiple control modes within a single material platform. They enable (i) interfacial anchoring and stabilization that suppress aggregation and structural degradation through multidentate interactions and steric confinement [55,56,57]; (ii) electronic and coordination modulation, in which functional groups tune charge distribution, interfacial dipoles, and coordination fields to reshape the binding energies of key intermediates [58,59,60,61]; (iii) microenvironment regulation, where polarity, fixed charges, and hydrogen-bond networks modulate local pH, ion activity, solvent organization, and reactant partitioning [62,63]; and (iv) transport management, in which morphology and ion-conducting pathways govern reactant/product diffusion and couple ionic and electronic fluxes [64,65,66]. Importantly, these layers can be made sufficiently thin and permeable to preserve site accessibility, while still providing robust, long-range control over interfacial chemistry.

**Figure 1:**
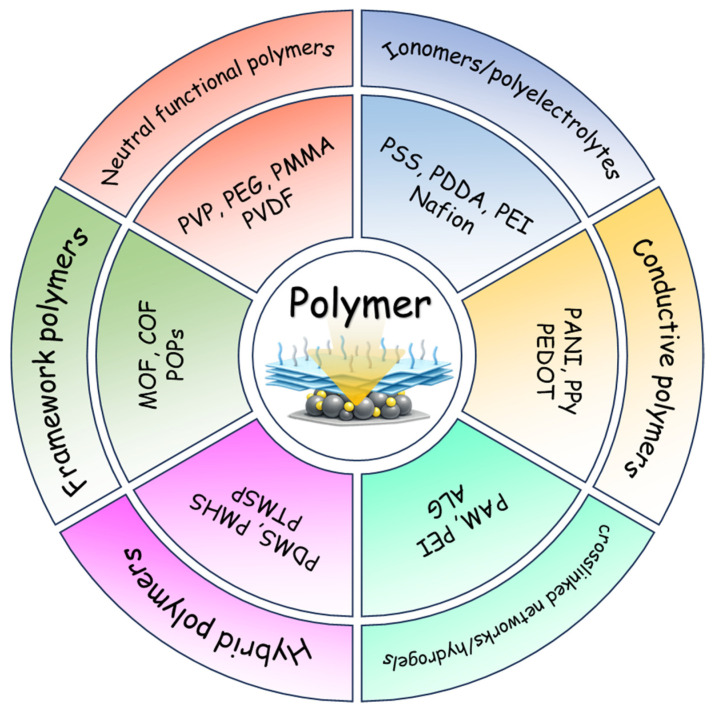
Overview of polymer platforms for nanocatalyst functionalization and representative examples. The schematic summarizes six classes of polymer interfaces—neutral functional polymers, ionomers/polyelectrolytes, conductive polymers, crosslinked networks/hydrogels, hybrid inorganic–organic polymers, and framework-type polymers (MOF/COF/POPs)—and highlights representative examples used to engineer catalytic interfaces.

To provide a clear, catalysis-relevant perspective, this review adopts a functional classification of polymer platforms rather than a textbook-style polymer taxonomy. Specifically, we organize polymer-functionalized nanocatalysts into six recurring material classes (Figure 1): (1) neutral functional polymers used as ligands, brushes, or thin shells to improve anchoring and interfacial wettability; (2) charged polymers and ionomers (polyelectrolytes) that create ion-selective environments and tune local pH and reactant activities; (3) conjugated and conductive polymers that couple interfacial chemistry with electronic transport; (4) crosslinked networks and hydrogels that act as confined microreactors, balancing stability and diffusion; (5) organoelement and inorganic–organic hybrid polymers that provide enhanced chemical robustness and selective permeability under harsh conditions; and (6) framework-type materials—including metal organic frameworks (MOFs), covalent organic frameworks (COFs), and porous organic polymers (POPs)—that introduce ordered porosity, site isolation, and molecular sieving as a “hard” form of polymer functionalization. Across these categories, we emphasize an evidence-driven structure–mechanism–performance chain. Key polymer parameters (e.g., thickness, coverage, charge density, conductivity, swelling, and porosity) should be linked to interfacial characterization and mechanistic readouts, and ultimately to catalytic metrics.

In this review, we first summarize the principal construction strategies, performance-relevant structural descriptors, and mechanistic concepts that underpin polymer-enabled functionalization. We then discuss six recurring polymer platforms, highlighting representative design motifs and clarifying how polymers stabilize active phases, reprogram interfacial microenvironments, and regulate transport. Finally, we compare performance trends across catalytic systems and identify key remaining challenges, including balancing protection with site accessibility, ensuring polymer integrity under harsh operating conditions, and standardizing the reporting of polymer descriptors to enable meaningful cross-study comparisons. Framing polymers as programmable interfacial materials, this review distills practical design principles for using polymer functionalization to deliver concurrent gains in activity, selectivity, and durability across nanocatalyst systems.

## 2. Construction Strategies and General Mechanisms of Polymer-Enabled Functionalization

Polymer functionalization can be achieved through diverse synthetic routes, but most approaches share a common goal: creating a polymer-defined interfacial region that is chemically addressable, structurally stable, and sufficiently permeable to preserve access to catalytic sites. In practice, the performance of polymer-functionalized catalysts depends not only on polymer chemistry but also on how the polymer is anchored and distributed at the surface, and on the interfacial descriptors that are tuned (e.g., thickness, coverage, charge density, swelling, porosity, and ionic/electronic conductivity). To facilitate cross-study comparison and to make the structure–mechanism–performance link more explicit, Table 1 summarizes the principal interfacial descriptors that are commonly tuned in polymer-functionalized catalysts and outlines how these descriptors map onto stabilization, microenvironment regulation, and transport management, as well as the resulting trends in activity, selectivity, and durability. The table also provides a cross-class perspective, highlighting the typical strengths and trade-offs of major polymer platforms under representative catalytic environments. Guided by this descriptor-based framework, the following section surveys representative construction strategies and distills the general mechanistic roles by which polymers functionalize nanocatalyst interfaces.

### 2.1. Construction Strategies

Polymer functionalization typically yields a limited set of interfacial motifs that largely determine which catalytic bottlenecks can be addressed. This motif-limited behavior reflects recurring physical constraints: how polymer chains attach to the surface (physisorption versus covalent grafting), how they pack and extend (isolated coils, brushes, or continuous films), and how much free volume and connectivity they leave for reactant access. As a result, polymer layers are not generic stabilizers but programmable interphases that can simultaneously regulate (i) catalyst stability, (ii) local solvation and polarity, (iii) reactant/product partitioning, and (iv) transport across multiple length scales [67]. These coupled effects determine whether the dominant performance gain arises from mitigating deactivation, shifting selectivity, or alleviating mass-transfer limitations [68,69,70]. In the light-touch regime, polymers behave as ligands or soft adlayers that adsorb on nanoparticle surfaces, tune interfacial wettability, and suppress aggregation. Beyond steric stabilization, these adlayers can modulate the local dielectric environment and hydrogen-bonding network, reorganize interfacial solvent structure, and shift the near-surface concentration of ions or polar substrates [68,71,72,73]. These effects are particularly important when rates are sensitive to proton-coupled steps, interfacial pH, or the availability of co-reactants such as water and hydroxide. Because adsorption is typically dynamic and patchy, light-touch motifs often preserve most active sites while selectively attenuating sites prone to overbinding, poisoning, or undesired side reactions [74]. In practice, this regime is best suited to bottlenecks related to colloidal stability and surface fouling, and to coarse tuning of hydrophilicity/hydrophobicity, rather than to strong molecular sieving or strict site isolation.

**Figure 2:**
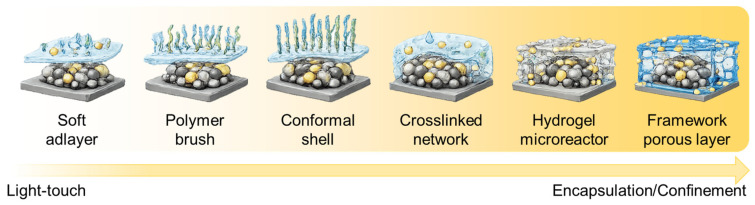
Interfacial motifs of polymer-enabled functionalization for nanocatalysts.

At higher structural definition, surface-tethered polymer brushes form an extended interphase whose thickness and composition can be tuned via chain length, grafting density, and solvent quality, enabling microenvironment control without fully encapsulating the active surface [75,76] (Figure 2). Brushes create a quasi-3D reaction zone where local polarity, viscosity, and ion permeability can be engineered, effectively decoupling interfacial conditions from the bulk. By adjusting brush parameters, one can tune the balance between accessibility and confinement. Dense brushes can enrich specific reactants through favorable interactions, exclude interferents via entropic/enthalpic penalties, and mitigate concentration polarization by providing continuous pathways for solvated species. Importantly, brush interphases can create gradient-like environments—for example, a highly hydrated, conductive outer region that transitions to a less polar inner region that favors adsorption of nonpolar substrates [77,78]. For instance, Toma et al. systematically compared polymeric and molecular modifiers with varying hydrophilicity/hydrophobicity, combining experiments with molecular dynamics simulations to correlate selectivity trends (e.g., H_2_/formate/CO) with surface-hydride stabilization and M–H bond strength modulation, thus illustrating how brush-derived polarity gradients directly influence reaction pathways [79]. This enables microenvironment engineering while avoiding the full diffusion penalties associated with thick encapsulation.

At the other end of the spectrum, conformal polymer shells and crosslinked networks form continuous architectures that provide stronger protection and longer-range transport pathways [80,81]. Conformal coatings can act as selectively permeable barriers that prevent leaching, corrosion, or fouling while still allowing reactants to access catalytic sites, provided the layer is sufficiently thin and porous. Here, thickness and porosity become first-order design variables. Ultrathin, defect-controlled films can suppress dissolution and mechanical detachment with minimal kinetic penalty, whereas thicker films improve durability at the cost of added diffusion resistance [82,83]. When properly engineered, conformal shells can mitigate local poisoning by slowing the ingress of strongly adsorbing species. They can also stabilize metastable surface states by buffering against aggressive chemical environments. In such cases, the polymer shell operates as a protective membrane whose selectivity is governed by solubility–diffusivity tradeoffs and the connectivity of free volume within the film.

Crosslinked networks and hydrogels embed catalysts within a swollen polymer matrix, effectively creating a microreactor that couples stabilization with local enrichment and diffusion control [84]. Unlike conformal shells, swollen networks can accommodate substantial solvent while maintaining mechanical integrity, enabling high permeability to selected species while suppressing aggregation and leaching over long timescales. The microreactor concept is especially powerful when performance is limited by coupled steps—such as sequential reactions, unstable intermediates, or competing pathways—because the network can tune residence time, local concentration, and co-reactant availability [85,86]. For example, functional groups within the gel can preconcentrate substrates through electrostatic or hydrophobic interactions, buffer local pH, or coordinate to metal centers to suppress restructuring [87]. Meanwhile, the chemical functionality of the network itself can impose selective transport rules, effectively creating a “chemical mesh”. For example, Bell et al. employed bilayer cation- and anion-conducting ionomer coatings on Cu [88]. Here, the ionomer layers function as chemically selective networks: anion-exchange ionomers increase the local CO_2_/H_2_O ratio via higher CO_2_ solubility and permeability, while cation-exchange ionomers raise local pH by capturing OH^−^ and excluding buffering carbonate species through Donnan exclusion. This programmed differential transport of key reactants, akin to a size- and charge-selective mesh, together improves C_2_^+^ selectivity.

### 2.2. Interfacial Attachment and Structural Descriptors

Attachment chemistry largely determines whether polymer functionalization remains intact under realistic catalytic conditions and constrains the interfacial architectures that can be realized [89,90]. Physisorption and supramolecular binding rely on electrostatic attraction, hydrogen bonding, π-π interactions, and hydrophobic association, offering synthetic simplicity and, in some cases, dynamic or self-healing interfaces [91,92,93]. However, because these interactions are sensitive to the local chemical environment, they can be weakened by changes in ionic strength, competitive adsorption, extreme pH, high potentials, or elevated temperatures. Such perturbations may trigger interfacial reorganization or partial desorption during operation.

**Figure 3:**
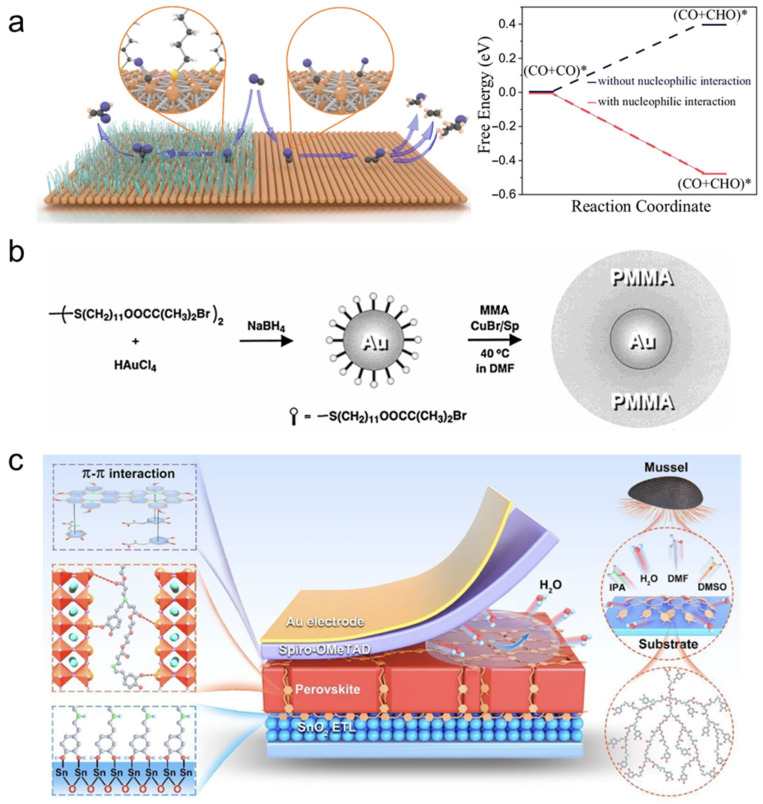
(**a**) Schematic illustration of how surface ligands influence product selectivity, together with geometry-optimized DFT results. The asterisk (*) denotes the adsorbed state and is a standard notation used in DFT cal-culations to indicate this specific status. Reprinted from Ref. [95] with permission from Nature Publishing Group. (**b**) Schematic representation of the synthesis of polymer-coated AuNPs via surface-initiated LRP. Reprinted from Ref. [96] with permission from ACS. (**c**) Schematic of the key components underlying underwater adhesion in natural mussels and the HPDA adhesive, and their roles in perovskite films and interfaces. Reprinted from Ref. [97] with permission from Nature Publishing Group.

More durable polymer–catalyst interfaces are generally achieved via coordination bonding, covalent anchoring, or in situ growth/encapsulation. Coordination and chelation—enabled by amines, pyridines, thiols, phosphonates, carboxylates, and related motifs—can provide multidentate attachment while simultaneously reshaping the local coordination field of surface metal sites [94]. Mechanistically, these interactions can stabilize reactive adsorbates, tune adsorption energetics through ligand-field/electrostatic effects, and, in some cases, create confined interfacial regions that increase the residence time and local activity of key intermediates [94]. A representative demonstration used octadecanethiol to build a deep-cavity microstructure on Cu particles, where confinement enriches carbonyl-related intermediates and promotes C–C coupling toward acetate; concurrently, the sulfur donor stabilizes carbonyl species through strong binding and nucleophilic interaction, lowering the kinetic barrier for coupling [95]. (Figure 3a) Despite these benefits, coordination-based motifs can still be vulnerable under electrochemical bias because changes in metal oxidation state and competitive adsorption by reactants, intermediates, or electrolyte ions may weaken ligand binding or reorganize the interface. Covalent grafting—via grafting-to or grafting-from routes—typically offers higher resistance to delamination and enables well-defined brushes with controlled coverage, but it comes with greater synthetic complexity and stronger dependence on surface chemistry, accessibility, and defect density.

Well-defined polymer brushes are most reliably obtained through surface-initiated grafting, which fixes chain density and minimizes desorption under operation [98,99]. Mechanistically, dense brushes stabilize nanoparticles by steric confinement and controlled solvation, while still allowing transport through the brush if chain length and grafting density are properly balanced. Using this approach, Poly(methyl methacrylate) (PMMA) was grown from nanocrystalline Au surfaces to form a dense brush layer via surface-initiated free-radical polymerization [96]. (Figure 3b) A complementary route is in situ polymerization or shell growth around nanoparticles, which can “lock” catalysts into intimate contact with a polymer matrix and suppress detachment or restructuring. The key trade-off is architectural: insufficient control can bury active sites, reduce permeability, or introduce tortuous diffusion paths that dominate the observed kinetics. A widely used adhesion platform employs polydopamine deposition, which provides substrate-agnostic interfacial binding and a versatile chemical handle for subsequent modification [100]. Although first established as a macroscopic coating strategy, the same catechol/amine adhesion motif has been broadly adapted to nanoparticle systems, where it enables conformal nanoshell formation and functions as an interfacial primer for secondary growth or post-functionalization. Across these approaches, a consistent design rule is to match attachment strength and interfacial architecture to the operating environment. Dynamic, weakly bound motifs can be beneficial when interfacial reorganization or self-healing is advantageous, whereas strongly oxidative, high-potential, or high-temperature regimes typically require robust anchoring and mechanically/chemically resilient architectures.

The same polymer chemistry can yield markedly different interfacial structures depending on how it is assembled, so quantitative descriptors are essential for building credible structure–performance relationships and avoiding overinterpretation of apparent activity gains [62]. The most informative descriptors include interfacial thickness and coverage (including uniformity), as well as functional-group identity and density, which collectively define coordination strength, fixed-charge density, and hydrogen-bonding capacity. For polymer brushes, chain length and grafting density are critical because they set interphase extension and the degree of local confinement [101]. In networked systems, crosslink density and swelling behavior determine the trade-off between mechanical robustness and permeability. For porous or framework-like coatings, effective porosity and tortuosity largely control site accessibility. Transport-related descriptors should be reported explicitly because they frequently dominate performance under realistic operating regimes. Ionic conductivity and ion-exchange capacity are central for polyelectrolytes and ionomers, whereas electronic conductivity is decisive for conjugated polymer layers and hybrid architectures intended to facilitate charge delivery. Finally, wettability metrics and reactant partitioning characteristics connect polymer polarity and microstructure to near-surface concentration fields, helping disentangle genuine intrinsic kinetic changes from improvements that primarily originate from altered mass transport, morphology, or microenvironment effects.

### 2.3. Mechanistic Functions and Design–Performance Linkages

Despite the diversity of polymer chemistries, their catalytic roles can be distilled into a small set of mechanistic functions that often operate simultaneously. A first, broadly applicable function is stabilization of active phases. Polymers can suppress nanoparticle migration, coalescence, and structural reconstruction through steric confinement and multidentate anchoring [102,103]. They can also reduce dissolution or leaching by forming protective, selectively permeable barriers. This stabilization route is often the most direct path to improved durability and retention of electrochemically or catalytically accessible surface area, particularly under harsh thermal or electrochemical conditions where bare nanostructures tend to sinter, corrode, or restructure [97] (Figure 3c). A second function is active-site and microenvironment regulation, which can reshape activity and selectivity. Coordinating groups and interfacial dipoles can shift charge distribution and the adsorption energetics of key intermediates. Fixed charges, dielectric screening, and hydrogen-bond networks can, in turn, influence local pH, ion pairing, solvent organization, and reactant partitioning near catalytic sites [104,105]. These effects are rarely single-parameter outcomes. Changes in functional-group chemistry typically alter both the energetic landscape (via coordination and electrostatics) and local concentrations (via partitioning and solvation), making microenvironment design a powerful but inherently coupled lever [106,107]. A third function is transport management, which becomes decisive in porous electrodes and high-rate regimes. Polymer morphology defines diffusion pathways for reactants and products and can couple ionic flux with electron delivery when ionomeric or electronically conductive domains are present. The practical implication is a familiar trade-off: polymer layers must be protective yet sufficiently permeable. Otherwise, diffusion barriers and concentration polarization can negate intrinsic kinetic gains and even invert apparent trends.

Accordingly, catalytic performance in polymer-functionalized systems is often dictated by a small set of polymer-controlled descriptors that act as practical interfacial “handles.” Activity can improve when polymers increase active-site utilization by suppressing aggregation, stabilizing reactive surface terminations, or facilitating coupled charge/ion delivery at the interface. Conversely, activity may decline when the layer becomes overly dense or thick, exhibits limited swelling, or lacks sufficient ionic/electronic conductivity, thereby restricting reactant access and product removal [108,109]. Selectivity is frequently the most polymer-sensitive metric because polymer polarity, fixed charges, and hydrogen-bonding motifs reshape the local chemical environment and alter the effective activities of reactants, protons, hydroxide, and spectator ions near catalytic sites [110,111]. Consequently, even modest changes in charge density, wettability, or microphase separation can substantially shift product distributions without changing the catalyst composition. Stability is typically enhanced when polymer architectures provide multidentate anchoring, mechanical buffering, and selective permeability that suppress leaching, corrosion, or surface reconstruction. However, long-term durability ultimately requires the polymer itself to remain chemically intact under operation; stability optimization therefore must consider both catalyst preservation and polymer degradation pathways [112,113]. Overall, the most transferable guideline is to treat polymer functionalization as a coupled optimization problem: thickness/coverage, charge density, swelling/permeability, and ionic/electronic conductivity should be tuned together to match the dominant limitation—intrinsic kinetics at low rates, microenvironment control for selectivity, or transport and durability under high-rate and long-duration operation.

## 3. Polymer Platforms for Functionalized Nanocatalysts

### 3.1. Neutral Organic Functional Polymers

Neutral organic functional polymers are among the most widely used and synthetically accessible platforms for nanocatalyst functionalization [114,115,116]. Their core advantage is the ability to form soft, conformal interphases through multidentate but typically non-ionic interactions, offering a practical balance between stabilization and active-site accessibility [117,118]. Because these polymers are solution-processable and readily tunable in molecular weight, segment polarity, and functional-group density, they provide a flexible handle to regulate nanoparticle dispersion, interfacial wettability, and the near-surface solvation environment without introducing strong long-range electrostatic fields [119]. As a result, neutral-polymer functionalization is especially effective when performance is limited by structural instability (aggregation, coalescence, sintering, or surface reconstruction), or when modest tuning of adsorption and interfacial mass transport is needed while maintaining broad compatibility across catalyst compositions and supports.

A defining feature of this class is its reliance on weak-to-moderate binding and dynamic interfacial organization, which often preserves site exposure and enables adaptive interfaces under operating conditions [120,121]. Mechanistically, neutral polymers stabilize dispersed nanophases via steric repulsion, reduce interparticle contact by buffering interfacial energies, and tune adsorption of reactive intermediates by reshaping the local dielectric environment and hydrogen-bond network adjacent to the surface. This “soft” stabilization, however, comes with an intrinsic trade-off: overly dense adsorption or excessive coating thickness can partially mask active sites and increase diffusion resistance, whereas insufficient coverage may fail to suppress restructuring under harsh conditions. Effective designs therefore emphasize controlled coverage and permeability, using polymer chemistry and architecture to retain dispersion and favorable interfacial conditions while minimizing transport penalties.

**Figure 4:**
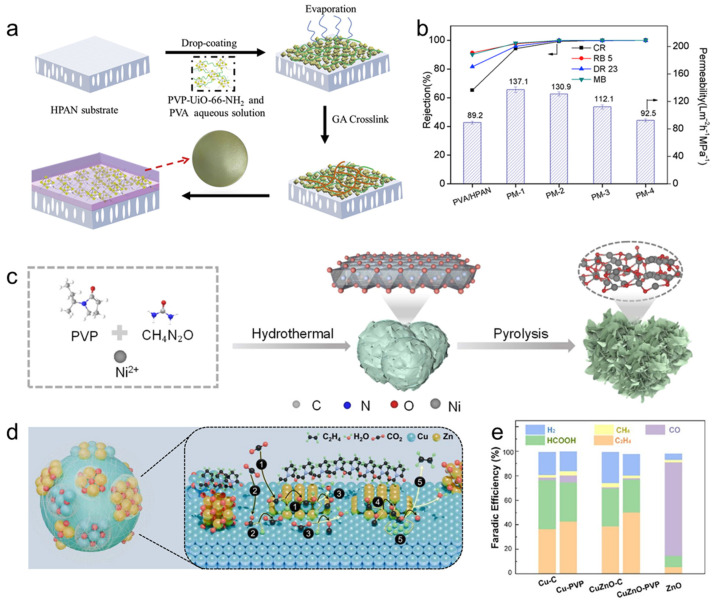
(**a**) Synthetic procedure for the PM membrane fabrication process. (**b**) Effect of PVP-UiO-66-NH_2_ concentration on the separation efficiency. Reprinted from Ref. [122] with permission from Elsevier. (**c**) Schematic illustration of the fabrication process for PVP-NiO_v_. Reprinted from Ref. [123] with permission from Elsevier. (**d**) Schematic representation for the reaction pathways of ECR on CuZnO-PVP surface. (Equations 1 and 2: CO_2_ adsorption, Equation 3: initial reduction, Equation 4: forming C-C bond, Equation 5: generate C_2_H_4_) (**e**) FEs of the products for Cu-C, Cu-PVP, CuZnO-C, and CuZnO-PVP. Reprinted from Ref. [124] with permission from Wiley-VCH.

In practice, the performance gains from neutral polymers are most often manifested as improved colloidal stability and interfacial compatibility, rather than long-range electrostatic effects. A representative example is polyvinylpyrrolidone (PVP) functionalization of UiO-66-NH_2_ nanofillers, where strengthened polymer–framework interactions enhance aqueous dispersibility and enable reliable downstream processing into robust nanocomposite thin films [122]. Notably, the absolute zeta potential increases from 31.1 mV (UiO-66-NH_2_) to 52.2 mV (PVP–UiO-66-NH_2_), consistent with a more stable colloidal state that resists aggregation over extended periods and supports reproducible film fabrication (Figure 4a,b). Beyond dispersion control, neutral polymers can also act as interfacial “directors” that bias surface terminations or defect populations, thereby reshaping adsorption geometry and intermediate binding without fully encapsulating active sites (Figure 4c). This role is particularly consequential when selectivity is dictated by binding configuration (e.g., end-on versus side-on adsorption). In oxygen electrochemistry, where O_2_ adsorption geometry strongly influences the 2e^−^ ORR pathway to H_2_O_2_, PVP-assisted oxygen-vacancy formation on NiO is reported to redirect O_2_ adsorption toward a Pauling-type configuration and increase the preference for the 2e^−^ route [123]. The resulting catalyst achieves ~95% H_2_O_2_ selectivity and a high H_2_O_2_ production rate of 15.12 mol g^−1^ h^−1^ in a flow-cell configuration, highlighting that neutral polymers can enhance performance by coupling defect regulation with adsorption/intermediate control rather than serving as passive binders.

Beyond dispersion control, neutral polymers can act as interfacial directors that reshape adsorption energetics by stabilizing specific surface terminations, defect populations, or hybrid junction motifs—often without fully encapsulating the catalytic surface. This role becomes particularly important when selectivity is dictated by binding configuration (e.g., end-on vs. side-on adsorption) or by the residence time of carbonyl/CO-like intermediates. In the PVP-modified NiO system discussed above, the polymer-assisted interfacial structure steers O_2_ adsorption toward a Pauling-type configuration, which favors OOH retention and thereby promotes the 2e^−^ ORR pathway [123]. A conceptually related but more structurally explicit strategy is to use neutral polymers to define and stabilize polymer–inorganic junctions that concentrate charge and enforce reaction-relevant adsorption states. Zhou and colleagues constructed a “polymer-enabled heterojunction” for CO_2_ reduction reaction (CO_2_RR) by inserting a few-atomic-layer ZnO bridge between Cu nanoparticles and a PVP overlayer (Figure 4d) [124]. Hydrogen-bond interactions associated with the N-vinylpyrrolidone motif stabilize the ultrathin ZnO layer and help confine electrons at the Cu/ZnO interface, leading to CuZn nanoalloy-like interfacial sites that synergistically enhance CO adsorption and increase the probability of C–C coupling toward C_2_H_4_. In addition, preferential binding of PVP to ZnO was proposed to suppress oxygen-adsorbate diffusion, inhibit Cu oxidation, and enrich CO_2_ in the vicinity of Cu. Together, these effects enabled a C_2_H_4_ selectivity of ~50.2% with stable operation over 10 h (Figure 4e).

A third recurring function is immobilization and device integration, where neutral polymers serve as compliant interlayers that anchor nanoparticles (or micro/nanostructured catalysts) onto supports, improving recyclability and resistance to material loss while keeping transport pathways open. The key design constraint is continuity versus accessibility: the polymer must be sufficiently continuous to prevent detachment or leaching, yet thin/porous enough to avoid burying active sites or throttling diffusion. This balance is illustrated by PVP-assisted assembly of PVDF/BiOBr photocatalytic membranes, which enabled uniform catalyst loading on fibrous supports and delivered strong wastewater-relevant performance (100% RhB degradation in 25 min; 93.8% tetracycline degradation in 30 min; 74.6% Cr^6+^ removal in 60 min), while maintaining durability (after five cycles, degradation remained >99% with only ~0.5% loss) [125]. Shi et al. provided a complementary example of polymer-enabled stability in aqueous environments, synthesizing PVP–FeMo_2_S_3_ and PVP–ZnMo_2_S_3_ nanocrystals with regular morphology and long-term stability [126]. They showed that PVP–ZnMo_2_S_3_ retained substantial antibacterial efficacy even after 20 recycling cycles, consistent with the broader principle that a neutral polymer corona can enhance processability, stability, and reusability without imposing excessive transport penalties.

### 3.2. Ionomers and Polyelectrolytes

Charged polymers and ionomers are particularly effective for catalyst functionalization because they do more than stabilize nanostructures—they actively program the interfacial microenvironment [127,128]. By introducing fixed charges and exchangeable counterions, these materials reshape the local electrostatic landscape and hydration structure, thereby tuning ion activities, ion pairing, and reactant partitioning in the immediate vicinity of catalytic sites. This capability is especially valuable in electrochemical and aqueous-phase catalysis, where apparent rates and selectivity are frequently governed by near-surface pH, ionic strength, and double-layer composition rather than by catalyst composition alone [129,130]. In porous electrodes, ionomers further act as ionic-transport architects, providing percolated ion-conduction pathways that couple ionic flux with electronic delivery under high current operation. A defining feature of this platform is its strong dependence on hydration and swelling, which simultaneously control microenvironment regulation and mass transport [131]. Polyelectrolyte layers can enrich or exclude charged species through Donnan-type partitioning, adjust local proton or hydroxide availability, and stabilize charged intermediates—effects that often translate directly into selectivity shifts among competing pathways. However, these benefits are not automatic: excessive ionomer loading or insufficient porosity can impose diffusion barriers and concentration polarization, whereas overly weak or highly swollen films may sacrifice mechanical integrity and interfacial stability during operation. Effective designs therefore co-optimize charge density, water uptake, film continuity, and permeability, so that microenvironment control is achieved without compromising reactant access or product removal.

**Figure 5:**
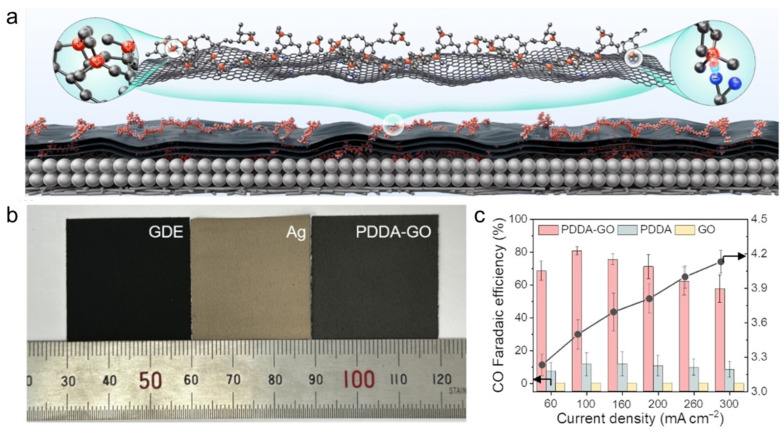
(**a**) Schematic illustration of the PDDA-GO modification layer assembled by electrostatic interactions. (**b**) Photos of (**left**) bare GDE, (**middle**) Ag/GDE and (**right**) PDDA-GO/Ag/GDE. (**c**) CO FEs of PDDA-GO-, PDDA- or GO-modified Ag catalysts at different applied current densities, together with the corresponding full-cell voltages of PDDA-GO-modified Ag. Reprinted from Ref. [132] with permission from Wiley-VCH.

A key mechanistic advantage of ionomers and polyelectrolytes is that their fixed charges and counterions allow the electric double layer and ion-pairing environment to be engineered directly at the catalyst surface, instead of being dictated by the bulk electrolyte. In CO_2_ electroreduction, this interfacial “cation effect” can accelerate key steps, yet it becomes problematic in acid-fed membrane electrode assemblies (MEAs) when mobile alkali cations accumulate and promote (bi)carbonate precipitation. One effective workaround is to replace free inorganic cations with immobilized organic cations that preserve a cation-like interfacial field while suppressing salt buildup. In this spirit, a poly(diallyldimethylammonium chloride) (PDDA)–graphene oxide assembly was used to confine tetraalkylammonium cations near the interface, displacing alkali cations but maintaining a favorable double-layer structure (Figure 5a,b). This design achieved a CO Faradaic efficiency (FEs) of 85%, a carbon efficiency of 93%, and an energy efficiency of 35% at 100 mA cm^−2^ in acidic operation, and it further enabled salt-free operation in pure water without precipitation or CO_2_ crossover (Figure 5c) [132]. More broadly, these results underscore a transferable rule for charged interphases: performance is governed by the coupled balance among charge density, film thickness, and proton/(bi)carbonate transport, rather than by polyelectrolyte loading alone.

**Figure 6:**
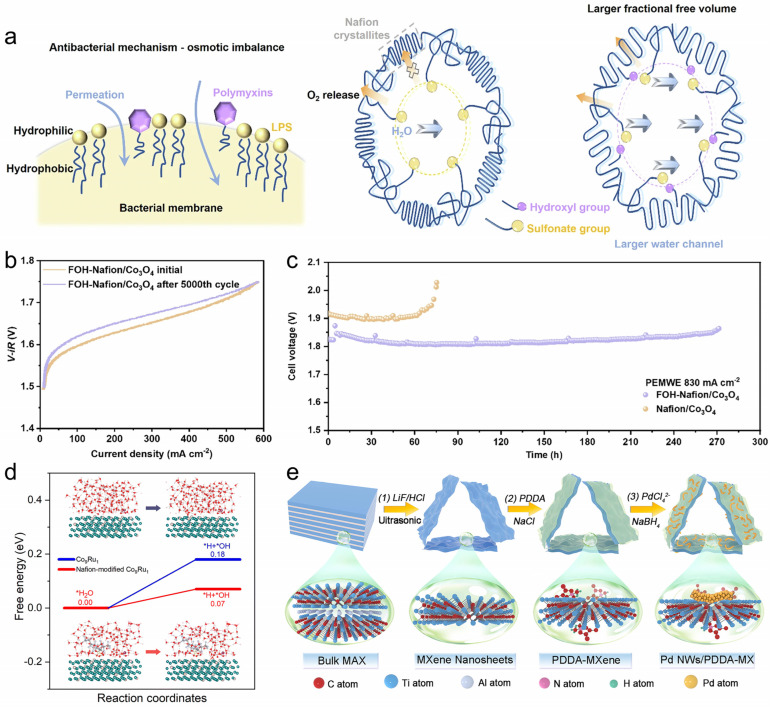
(**a**) Schematic illustration of the PDDA-GO modification layer assembled by electrostatic interactions. (**b**) Current-voltage polarizations of the PEMWE after 5000 multiple-voltage cycles of FOH-Nafion/Co_3_O_4_. (**c**) Chronopotentiometry test of FOH-Nafion/Co_3_O_4_ and Nafion/Co_3_O_4_ at 830 mA cm^−2^. Reprinted from Ref. [133] with permission from Nature Publishing Group. (**d**) Water dissociation pathways on bare and Nafion-modified Co_9_Ru_1_. Reprinted from Ref. [135] with permission from The Royal Society of Chemistry. (**e**) Illustration of the synthetic processes for Pd NWs/PDDA-MX electrocatalyst. Reprinted from Ref. [136] with permission from Elsevier.

At device-relevant current densities, ionomers become transport architects: they define the morphology of water/ion pathways, control wetting, and govern how effectively nanoparticle surfaces are accessed inside porous catalyst layers. The core mechanistic link is microstructure-to-transport coupling—seemingly minor changes in ionomer packing can switch the rate-limiting regime from interfacial kinetics to mass transfer. One illustrative strategy is molecularly modifying Nafion with a fluorinated alcohol dopant (CF_3_CF_2_CF_2_CH_2_OH) (Figure 6a) [133]. Through specific interactions with sulfonate groups (often described as oxonium-salt-like pairing) and preferential partitioning into polytetrafluoroethylene (PTFE)-like domains, the dopant disrupts ordered chain packing and increases free volume, thereby enhancing water-channel connectivity. Therefore, both charge-transfer and mass-transfer resistances are reduced, enabling sustained high-rate operation (about 1.81 V for 270 h at 830 mA cm^−2^) (Figure 6b,c). The same interphase also mitigates cobalt leaching, highlighting a general design principle: hydration-state control and transport optimization can translate directly into durability gains, not only activity improvements.

Even when a charged polymer is introduced nominally as a binder, it can become a first-order mechanistic lever by reshaping interfacial solvation, adsorption statistics, and local reactant activities. In CO_2_RR on Cu nanoparticles, Nafion decoration has been shown to increase surface hydrophobicity, which elevates the near-surface CO_2_ concentration and effectively raises the local CO_2_ activity [134]. Beyond partitioning, fluorine-rich motifs were further proposed to contribute an additional CO_2_-activation component through specific acid–base-like interactions, collectively accelerating *CO formation and increasing the probability of C–C coupling. These coupled effects were correlated with a C_2_ FEs of 73.5% at −1.2 V vs. RHE while suppressing HER. A parallel logic applies to nitrate reduction, where Nafion can tune the proton supply and substrate binding in a coordinated way: mechanistic analysis on a Co_9_Ru_1_ model suggests that Nafion facilitates water dissociation (as a proton source) and strengthens NO_3_^−^ adsorption through interfacial orbital interactions with *NO_3_, thereby improving nitrate delivery to the reactive plane without strongly promoting HER (Figure 6d) [135]. Beyond solvation and adsorption, polyelectrolytes can also impose directional charge-flow control in hybrid interfaces. An ultrathin insulating PDDA interlayer has been shown to act as an electron-withdrawing and relaying mediator between a semiconductor and different cocatalyst “electron reservoirs,” accelerating interfacial charge-transfer kinetics across multiple materials pairings (Figure 6e) [136]. In conclusion, by co-tuning partitioning, adsorption energetics, and charge-transfer topology, they can redirect both rate and selectivity—yet only when thickness, hydration, and transport resistances are kept in a regime that does not penalize flux.

### 3.3. Conductive Polymers

Conjugated (conductive) polymers offer a distinctive advantage in polymer-enabled functionalization because they can simultaneously tune interfacial chemistry and conduct charge [137]. When integrated with nanocatalysts, they can form electronically percolated networks that enhance charge delivery to dispersed sites, while their functional groups tune adsorption, local polarity, and wettability. This dual role is particularly valuable in electrocatalysis and photoelectrocatalysis, where interfacial charge transfer and local electric fields strongly govern reaction kinetics. In addition, polymer layers can serve as protective skins that mitigate corrosion and suppress detachment of active phases [138]. The performance of conductive-polymer-functionalized catalysts is often dictated by the polymer’s doping state, electrical conductivity, and redox stability under operating conditions. With appropriate architectural control, these layers can reduce interparticle contact resistance, buffer mechanical stress, and redistribute the interfacial potential drop, thereby improving apparent activity and stability. At the same time, they introduce clear trade-offs: thick coatings can introduce diffusion limitations, and some conductive polymers may undergo over-oxidation, dedoping, or chemical degradation under harsh potentials or reactive environments. Consequently, thin, porous, and robustly anchored conductive layers are generally preferred because they preserve charge transport while maintaining access to catalytic sites.

**Figure 7:**
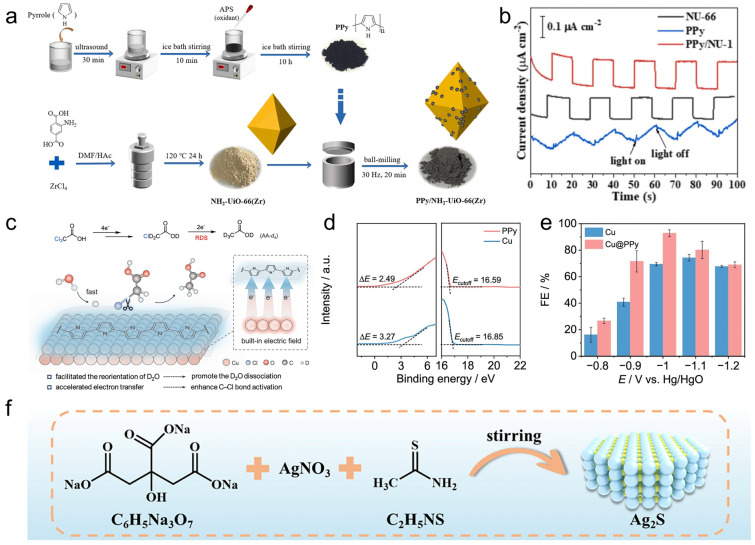
(**a**) Schematic representation of PPy/NU-66. (**b**) Transient photocurrent responses. Reprinted from Ref. [139] with permission from Elsevier. (**c**) Strong built-in electric field of the PPy-modified Cu electrode. (**d**) UPS spectra of Cu and PPy. (**e**) FEs of AA against applied potential over Cu@PPy and Cu. Reprinted from Ref. [140] with permission from ACS. (**f**) Preparation process of catalyst Ag_2_S/PPy/CA. Reprinted from Ref. [141] with permission from Nature Publishing Group.

Conductive polymers can operate as interfacial regulators of both electron delivery and mass transport, rather than serving merely as binders. When a thin conjugated overlayer is electronically percolated, it can wire dispersed active sites and smooth local potential drops, while its polarity, free volume, and segment packing reshape solvation structure and gas–liquid partitioning near the reaction plane. This combination enables a “conformal-but-permeable” skin that simultaneously enriches the target reactant at the interface, suppresses competing pathways by tuning wetting and adsorption statistics, and mitigates corrosion or particle detachment without fully screening active sites. One illustrative strategy employs an N-rich polypyrrole (PPy) shell on Ag nanoparticles, where the polymer is proposed to increase local CO_2_ availability and stabilize an interfacial environment less favorable for proton access and H_2_ evolution (Figure 7a) [139]. Consistent with this microenvironment-driven picture, PPy coating shifts the surface from hydrophilic to hydrophobic (contact angle increasing from 51.50° to 94.97), markedly suppressing H_2_ formation. As a result, CO selectivity rises from 43.8% to 91.7%, while H_2_ selectivity decreases from 52.9% to 6.1% under identical conditions, highlighting how conductive-polymer skins can steer selectivity by coupling interfacial partitioning with transport and stability control (Figure 7b).

Beyond microenvironment tuning, conductive polymers can generate intrinsic electric fields at metal–polymer junctions that accelerate interfacial charge transfer and reorient polar molecules at the reaction plane—an advantage when the rate-limiting step is field-sensitive bond activation or tightly coupled proton–electron transfer. In a Cu–PPy heterointerface, a work-function mismatch induces spontaneous charge redistribution and establishes a built-in electric field pointing from Cu to PPy (Cu: 4.37 eV; PPy: 4.63 eV; delta = 0.26 eV) (Figure 7c,d) [140]. This junction field is accompanied by measurable electron transfer (reported as 0.29 e from Cu to each pyrrole unit), which effectively increases electronic coupling between the surface and adsorbates and lowers the barrier for D_2_O dissociation, thereby boosting the interfacial supply of *D/*H equivalents that feed the key elementary step. These interfacial-field effects translate directly into performance, reducing the required overpotential by ~100 mV and delivering 94% FEs for the target product, with scalability also demonstrated (Figure 7e). More broadly, the same junction-engineering logic carries into semiconductor-like architectures: PPy can function as a p-type component that promotes directional carrier separation in polymer–inorganic composites. For instance, integrating PPy with NH_2_-UiO-66 to form a direct Z-scheme heterojunction has been used to suppress recombination by leveraging PPy’s carrier mobility and band alignment.

A third recurring role is defect/active-center engineering coupled with conductive-network construction. In this mode, the polymer does more than improve wiring and wetting: it creates or stabilizes catalytically productive defects (e.g., anion vacancies) and then preserves their kinetic advantage by maintaining an interfacial state with low charge- and mass-transfer penalties. A representative ternary Ag_2_S/PPy/carbon-aerogel architecture demonstrates this coupling [141]. Here, PPy promotes the formation of sulfur vacancies (VS), while the PPy skin together with the 3D carbon aerogel establishes continuous electronic pathways and open transport channels, jointly lowering impedance and mitigating concentration polarization (Figure 7f). As a result, CO selectivity remains at ~90% or higher over a wide potential window, with a peak value of 94.5%. 17 Mechanistically, in situ spectroscopy associates VS with enhanced electron uptake by the *COOH intermediate on the pathway to *CO, indicating that the vacancy sites selectively stabilize/activate the key branching intermediate rather than merely increasing surface area.

### 3.4. Crosslinked Networks and Hydrogels

Crosslinked networks and hydrogels function as polymer microreactors that immobilize nanocatalysts while enabling controlled transport through a swollen, porous matrix. Their key advantage is the ability to combine structural stabilization with microenvironment regulation [142,143]. The network can suppress nanoparticle migration and coalescence, buffer mechanical or chemical stress, and simultaneously create a hydrated interphase that governs reactant enrichment, local ion distribution, and intermediate stabilization. Because crosslink density, functional-group content, and water uptake are tunable over wide ranges, these materials can be adapted to catalysts that require long-term structural integrity or operation in complex, impurity-containing feeds. The central design variable in this class is the balance between confinement and permeability. Higher crosslink density typically improves mechanical robustness and resistance to leaching, but it can reduce swelling, slow diffusion, and limit effective site accessibility [144,145]. Conversely, highly swollen gels can offer low transport resistance and strong microenvironment effects, yet they may suffer from poor mechanical stability or dynamic restructuring under flow and potential cycling. In practice, successful hydrogel functionalization often relies on hierarchical porosity and robust anchoring points, allowing the gel to stabilize the catalyst while still supporting rapid reactant and product flux.

Crosslinked polymer networks and hydrogels can be viewed as microreactors that merge structural stabilization with microenvironment control. Unlike thin adlayers, a swollen network creates a water-rich yet structured interphase that (i) immobilizes nanophases and buffers mechanical/chemical stress, (ii) reshapes local concentration fields through hydrogen-bonding/coordination sites, and (iii) regulates diffusion via hydrated but tortuous transport pathways. This architecture is most beneficial when performance is limited less by intrinsic surface kinetics and more by intermediate-triggered deactivation, local pH drift, or long-term mass-transport instability. A recurring design rule is to avoid “burying” the active surface by introducing hierarchical porosity—macrochannels for electrolyte replenishment coupled with a hydrated nanoscale mesh for local regulation—so protection and microenvironment tuning are gained without prohibitive diffusion penalties.

A representative “hydrogel-as-soft-armor” strategy stabilizes nitrate-to-ammonia electrocatalysis on FeOOH nanowire arrays using a polyacrylamide (PAM) hydrogel [146]. The key mechanistic idea is intermediate management: strongly reducing intermediates, particularly NH_2_OH, can chemically reduce Fe(III) and accelerate catalyst failure, whereas the amide-rich PAM matrix hydrogen-bonds with NH_2_OH and biases its residence away from the FeOOH surface while still allowing downstream conversion to NH_3_. Crucially, ordered vertical channels introduced by ice templating preserve electrolyte access and prevent the coating from becoming a diffusion-blocking skin. With this channeled hydrogel interphase, the electrode delivered an NH_4_^+^ production rate of 8.64 mg h^−1^ cm^−2^ and a peak FEs of 81.20%, while maintaining high selectivity (about 85.77–82.10%) over 60 h of cycling.

**Figure 8:**
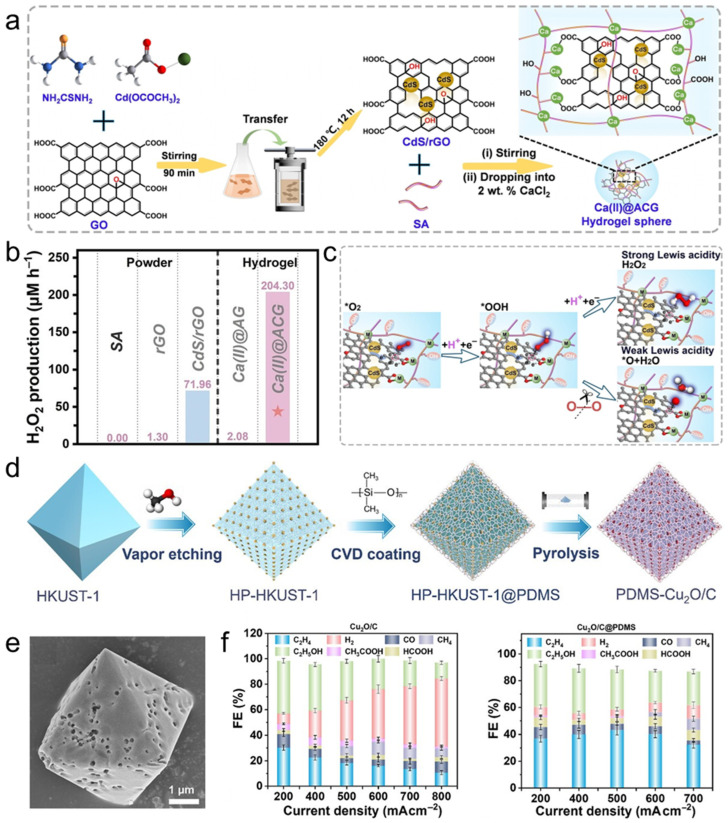
(**a**) Scheme of the synthetic procedure for the Ca(II)@ACG hydrogel spheres. (**b**) Photocatalytic performance for H_2_O_2_ production. (**c**) Illustration of the ORR reaction pathway on M-rGO. Reprinted from Ref. [147] with permission from Wiley-VCH. (**d**) Synthesis, morphology, and pore and surface structure of the catalysts. (**e**) SEM image of PDMS-Cu_2_O/C. (**f**) The measured FEs of PDMS-Cu_2_O/C and Cu_2_O/C@PDMS. Reprinted from Ref. [148] with permission from Wiley-VCH.

Hydrogels can also accelerate proton-coupled pathways by functioning as local proton relays and by tuning the residence time of key adsorbates. A metal-ion-crosslinked alginate–graphene hydrogel microreactor has been used to promote selective 2e oxygen reduction to H_2_O_2_ in pure water (Figure 8a) [147]. In their proposed mechanism, carboxyl and hydroxyl groups in alginate create a proton-rich environment that accelerates *OOH formation, while O=C–O–M(n) crosslinks at graphene edges modulate charge distribution to weaken overly strong *OOH binding and promote timely peroxide desorption. This suppresses O–O bond cleavage that would otherwise favor the four-electron pathway. This proton-relay plus controlled-desorption design delivered high H_2_O_2_ production (204.3 μM) and a mass-normalized production rate of 1021.5 μmol g_cat_^−1^ h^−1^ for Ca(II)-crosslinked hydrogel spheres in O_2_-saturated pure water (Figure 8b,c). More broadly, these results align with a recurring design rule for polymer–catalyst interfaces: amine- or carboxyl-rich domains can act as local proton reservoirs and relays, a central motif in proton-coupled electrocatalysis of polymer-functionalized metal nanocrystals.

### 3.5. Hybrid and Inorganic-Organic Polymers

Organoelement and inorganic–organic hybrid polymers extend polymer functionalization into harsher operating windows by combining organic interfacial programmability with inorganic-like robustness [149,150]. Backbones based on siloxane, phosphazene, and sol–gel-derived hybrid networks can form mechanically resilient, chemically tolerant interphases that suppress corrosion, oxidation, and dissolution while remaining permeable enough to sustain catalysis. The catalytic value of these hybrids rarely comes from “protection” alone; instead, they act as selective, dynamic barriers whose permeability and surface energetics can be tuned so that aggressive species are slowed while target reactants remain transport-accessible [151]. The design challenge is therefore inherently coupled: interfacial adhesion and selective permeability must be optimized together. Overly dense or poorly swollen layers impose diffusion penalties and lower apparent activity, whereas loosely crosslinked or highly porous films sacrifice protection and erode stability gains. Mechanical mismatch can further complicate long-term durability, especially when inorganic content and crosslink density increase brittleness under potential cycling, thermal gradients, or flow perturbations. Consequently, effective hybrids tend to be thin, conformal, and defect-tolerant, with transport pathways intentionally engineered rather than treated as incidental free volume.

Hybrid (inorganic–organic) polymers—especially siloxane-based materials—are attractive because they combine polymer-like interfacial programmability with inorganic-like robustness. Mechanistically, they often operate by (i) forming a chemically tolerant barrier that resists oxidation, corrosion, and delamination; (ii) tuning wettability and gas–liquid partitioning to reshape local reactant coverage; and (iii) introducing soft yet persistent interfacial dynamics that suppress fouling without fully blocking active sites. A key advantage is that these coatings can be covalently tethered for durability while retaining a liquid-like surface character that supports transport, helping to mitigate the classic protection–permeability trade-off.

Siloxane interfaces illustrate how hybrid polymers can operate as microenvironment levers rather than inert coatings. When covalently tethered, Polydimethylsiloxane (PDMS)-like layers can preserve adhesion while retaining a “liquid-like” interfacial character that supports transport, partially easing the classic protection–permeability trade-off. In electrochemical C–C coupling, a key mechanistic motif is the ability of siloxane layers to decouple C–C formation from hydrogenation/H_2_ evolution by reshaping both surface coordination and interfacial water organization. For example, constructing a covalently elaborated PDMS–Cu interface with a defined Si–O–Cu motif biases Cu toward ethylene formation (Figure 8d,e), reaching an ethylene FEs up to 71% and a partial ethylene current density of 513.6 mA cm^−2^, accompanied by a higher ethylene-to-ethanol ratio (Figure 8f) [148]. This behavior is consistent with interfacial covalent modulation that alters the local coordination/electronic landscape of Cu and thereby stabilizes the intermediate ensemble leading to ethylene while disfavoring competing hydrogenation routes. A complementary mechanistic lens emerges from PDMS-modified Cu(111), where increased hydrophobicity reorganizes interfacial water into a more strongly hydrogen-bonded structure with slower reorientation and a longer metal–H distance, suppressing the Volmer step and reducing H_2_ formation while increasing C_2_^+^ selectivity. Together, these perspectives highlight a unifying point: hybrid siloxane layers can steer selectivity by simultaneously tuning adsorption energetics and field-/water-mediated proton delivery at the reaction plane.

Beyond electrochemistry, hybrid siloxane coatings can stabilize catalysts under harsh feeds by actively managing fouling and interfacial activation, not merely by blocking poisons. Anchoring PDMS on CrO_x_/Al_2_O_3_ has been proposed to create a surface with “cilia-like” chain dynamics that continuously perturb and displace C_4_–C_6_ oligomeric precursors, suppressing carbonaceous deposition without introducing severe steric blocking of desired pathways [152]. In another direction, PDMS-containing composites can couple mechanical motion to interfacial charge effects: repeated contact–separation can generate non-uniform surface charges and transient local electric fields that enrich electrons and promote reactive oxygen species formation, enabling near-complete formaldehyde removal with markedly improved CO_2_ conversion efficiency relative to uncharged controls [153]. These examples emphasize that the “hybrid advantage” is not limited to chemical robustness; it also lies in enabling dynamic, defect-tolerant interfaces that reshape transport, local fields, and deactivation chemistry under realistic operating perturbations. Overall, hybrid (inorganic–organic) polymers are most effective when treated as engineered interphases—where covalent adhesion, selective permeability, interfacial wetting/water structure, and dynamic chain motion are co-designed—so that stability gains are achieved without turning transport into the dominant limitation. This same design logic naturally connects to framework-type coatings, where robustness and selectivity are likewise determined by a deliberately constructed transport landscape.

### 3.6. Framework Polymers

Framework-type polymeric materials can be regarded as a “hard” mode of polymer-enabled functionalization, in which porosity and topology become explicit design variables rather than incidental microstructure. When deployed as coatings, shells, or scaffolds around nanocatalysts, MOFs, COFs, and POPs impose an engineered transport landscape: ordered or semi-ordered pores can enrich target reactants, isolate active centers, and introduce size- or shape-selective flux that suppresses undesired side reactions [154,155,156]. This is especially valuable for stabilizing highly dispersed species, including single atoms and sub-nanometer clusters, because framework nodes and linkers offer well-defined anchoring environments that resist migration and aggregation while keeping access pathways open. The same structural rigor also creates nontrivial integration constraints [157,158]. If the framework layer becomes too thick or too dense, diffusion resistance can dominate and mask intrinsic kinetics; if catalyst–framework contact is incomplete, charge transfer can become the bottleneck in electrocatalysis [159,160]. In addition, framework stability is strongly condition-dependent: hydrolysis, ligand exchange, and partial collapse can occur in aggressive electrolytes or reactive atmospheres, and some linkers or metal nodes may undergo redox-driven degradation, undermining long-term operation [161]. From a practical perspective, advanced framework systems can also face cost and scalability constraints, arising from multistep syntheses, solvent- and time-intensive crystallization, and in some cases the use of expensive linkers or metal precursors; these factors can limit large-area electrode integration and translation to device-relevant manufacturing [162]. Effective framework functionalization therefore tends to emphasize thin or hierarchical architectures, robust interfacial coupling to the catalytic phase, and pore chemistries tuned to balance selectivity control with efficient mass transport, while prioritizing chemically robust nodes/linkers and more scalable synthetic routes for realistic deployment.

**Figure 9:**
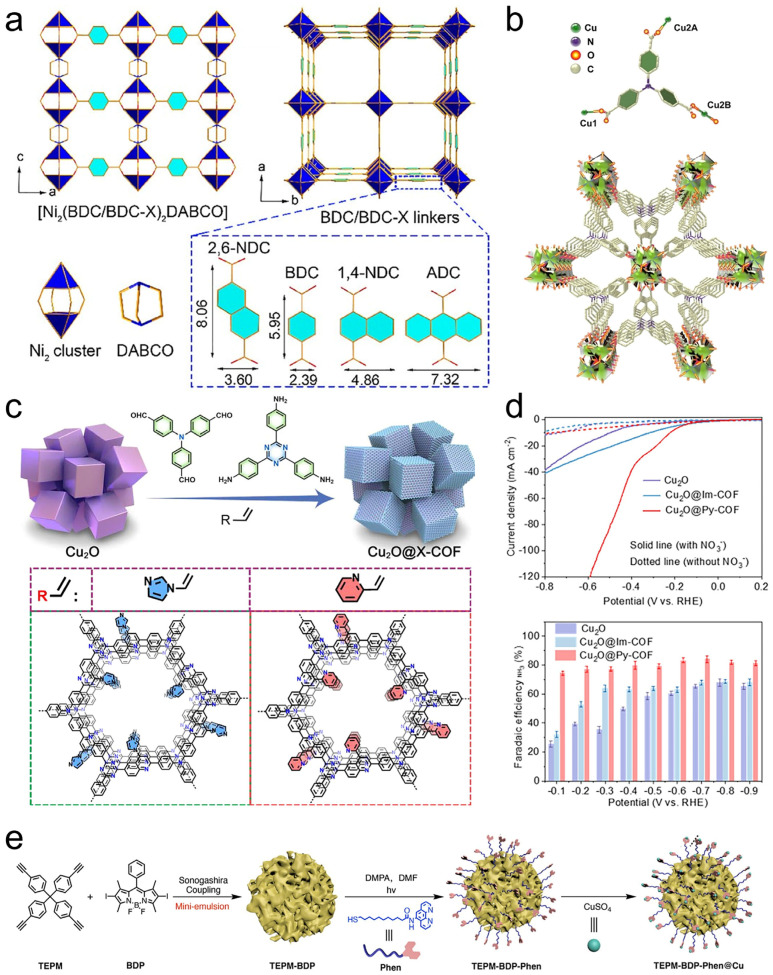
(**a**) Top and bottom views on the porous DMOF-type structures along the c-axis. Reprinted from Ref. [163] with permission from Wiley-VCH. (**b**) The coordination environments of Cu-TCA and the 3D framework of Cu-TCA along the *α*-axis. Reprinted from Ref. [164] with permission from Wiley-VCH. (**c**) Synthesis and characterization of core–shell Cu_2_O@COFs structures. (**d**) LSV curves and potential-dependent FE. Reprinted from Ref. [168] with permission from ACS. (**e**) Synthesis of TEPM–BDP under mini-emulsion conditions and subsequent post-modification to afford TEPM–BDP–Phen and TEPM–BDP–Phen@Cu. Reprinted from Ref. [170] with permission from ACS.

A useful way to view framework coatings is as hard interphases that add geometric control on top of chemical functionality. Their pores define a size- and shape-selective transport field, so the dominant lever is often partitioning: which species are concentrated near the active site, which are excluded, and how long intermediates are retained before desorption or further reaction. In this sense, pore descriptors such as aperture, connectivity, and local surface polarity become catalytic parameters because they directly set the balance between adsorption thermodynamics and diffusion kinetics. One illustration is systematic linker modulation that compresses or expands micropores in the ~4–9 Å regime, enabling an “optimal match” between channel dimensions and the kinetic diameter of the target molecule and thereby improving capture/selectivity with robust cycling behavior (Figure 9a) [163]. Beyond tuning rates by transport matching, frameworks can steer pathways by selectively admitting only a subset of substrates into the catalytic microenvironment. A Cu–TCA framework with 1D channels and a rhombic window of 6.5 Å × 6.5 Å exemplifies this logic: the aperture blocks bulky phosphine oxide while allowing CO_2_ and propargylic amines to access the interior, effectively filtering out a competing coordination route and funneling the multicomponent reaction toward a single productive pathway (Figure 9b) [164]. In practice, the “selectivity gain” here is not only steric exclusion; it is also kinetic, because restricting co-localization of bulky inhibitors preserves productive site occupancy and reduces time spent in off-cycle states. A third mechanism that translates well to nanoparticle functionalization is controlled reconstruction. Frameworks can operate as structured precursors that evolve into catalytically competent phases in a spatially regulated manner, while the inherited architecture preserves percolation and mitigates mechanical degradation during long operation. A pore-partitioned MOF platform that reconstructs into active oxyhydroxide domains while sustaining 1000 mA cm^−2^ for 200 h at ~1.9 V captures this concept and highlights how “stability” can arise from orchestrated, rather than accidental, phase evolution [165].

Compared with MOFs, COFs provide an all-organic (or predominantly organic) crystalline scaffold in which ordered channels and programmable functional groups form a molecular-gate microenvironment around nanoparticle surfaces [166,167]. The design advantage is the ability to couple transport selectivity (channel geometry) with chemical mediation (H-bond donors/acceptors, ionic sites, dipoles), thereby tuning proton/electron delivery and stabilizing specific adsorbate states without fully burying the active interface. A Py-COF shell grown on Cu_2_O nanocubes illustrates this “gate” concept in nitrate-to-ammonia electrocatalysis (Figure 9c), delivering an NH_3_ partial current corresponding to 2.3 mg h^−1^ cm^−2^ with ~84% FEs and stable operation for ~40 h (Figure 9d) [168]. Importantly, the COF thickness behaves like a first-order knob: an optimized thickness of ~35 nm balances confinement (to stabilize key surface intermediates and shape the local reaction environment) against accessibility (to avoid diffusion throttling and ensure charge delivery). COFs also provide a clean platform to show pore size as a kinetic control variable: within isoreticular designs, enlarging pores can increase CO formation rates in photocatalytic CO_2_ reduction while maintaining high selectivity, consistent with pore-governed changes in residence time and local reactant availability rather than changes in the intrinsic catalytic center.

Porous organic polymers lie at the “practical” end of framework polymers [169]. Although often amorphous or semi-ordered, they can be easier to synthesize, process, and integrate as robust coatings or supports on nanoparticles. Mechanistically, POP layers are effective when the goal is to combine high site density (many anchoring motifs per mass/area) with continuous yet permeable transport through micro/mesoporous free volume, enabling immobilization of molecular-like active centers, reduced leaching, and catalyst recycling. A Cu(II) surface-functionalized POP platform (TEPM-BDP-Phen@Cu) provides a representative case: Cu sites embedded in a porous organic polymer architecture deliver heterogeneous one-pot sulfoxide synthesis (Chan–Lam-type coupling) with yields up to 78%, leveraging confinement for selectivity while maintaining sufficient accessibility for turnover (Figure 9e) [170]. Overall, framework-type polymer functionalization is most powerful when pores are treated as quantitative catalytic handles: aperture/connectivity/polarity and shell thickness/interfacial coupling should be co-designed so that selectivity gains from enrichment/exclusion are achieved without allowing diffusion or charge transfer to become the dominant limitation.

### 3.7. Cross-Platform Comparison and Selection Guidance

Although the six polymer platforms are described separately, their real-world performance usually depends on how each balances three coupled interfacial functions: stabilizing the catalyst, regulating the local microenvironment, and managing mass and charge transport. In practical catalytic environments—especially under high-flux, device-level operation or chemically harsh conditions—measured activity, selectivity, and durability are often governed by whichever function becomes rate-limiting or triggers failure [171,172,173]. Therefore, selecting among platforms is most effective when guided by the dominant bottleneck rather than polymer identity alone. Relevant bottlenecks include interfacial ion activity and double-layer composition; diffusion and concentration polarization in porous layers; electronic percolation and potential drop; and resistance to corrosion or fouling.

Neutral polymers can serve as a broadly compatible, “soft” interphase when the main goal is to suppress aggregation, coalescence, or restructuring while maintaining accessibility [174]. They stabilize catalysts through multidentate, typically nonionic interactions and by tuning local solvation and wettability, without imposing strong long-range electrostatic fields. By contrast, ionomers and polyelectrolytes are often preferred when performance is controlled by near-surface ion activity, local pH gradients, and double-layer composition [175]. Their fixed charges and associated counterions can tune ion partitioning and interfacial electrostatics, thereby steering competing pathways through microenvironment control. However, this benefit is conditional: the charge density and hydration that improve microenvironment regulation can also increase swelling and create diffusion barriers if the polymer phase becomes too continuous or too thick, highlighting the need to co-optimize accessibility and mass transport.

In porous electrochemical electrodes, ionomers often excel when performance is limited by ionic polarization and reactant delivery at high current densities because they can form percolated ionic pathways that couple local ion-activity regulation with sustained ion transport throughout catalyst layers [176,177]. By contrast, hydrogels and crosslinked networks are most effective when the bottleneck involves intermediate management, residence-time control, or deactivation chemistry; in these cases, a microreactor-like environment that stabilizes key intermediates, buffers local chemical fluctuations, or suppresses detrimental side reactions can matter more than maximizing ionic conductivity [178]. The same distinction explains why channel-engineered gels can perform well without forming diffusion-blocking “skins”: hierarchical porosity and robust anchoring enable gels to provide confinement-driven microenvironment effects while preserving flux. In practice, ionomers are commonly chosen for transport- and polarization-limited regimes, whereas hydrogels are chosen for microenvironment- and deactivation-limited regimes, if permeability and mechanical integrity are engineered for the relevant operating window.

At high current densities, thick catalyst layers and porous architectures often suffer from local potential drops, contact resistance, and nonuniform current distribution [179,180]. In these regimes, conductive polymers can be advantageous because electronically percolated networks improve charge delivery to dispersed active sites while maintaining interfacial chemical tunability [181]. Their advantage is most evident when electronic wiring and interfacial charge transfer limit the apparent kinetics, provided that the doping state and oxidative stability remain compatible with the operating potential window. By contrast, inorganic–organic hybrid polymers are often chosen when the dominant constraint is chemical robustness under harsh conditions (e.g., corrosive media, aggressive potentials, and thermal or flow perturbations). Hybrid backbones can provide barrier-like protection and improved tolerance while still enabling selective permeability and wettability control. The key trade-off is coupled: hybrids that maximize protection through dense networks can introduce transport limitations and interfacial resistance, whereas designs that preserve permeability and minimize resistance translate robustness into practical durability without sacrificing rate.

Framework-type coatings (MOFs, COFs, and POPs) are particularly advantageous when performance is governed by pore-defined enrichment/exclusion and molecular-scale confinement [182]. In this setting, they enable size- and shape-selective flux, controlled intermediate residence times, and stabilization of highly dispersed active centers. Their advantage is most evident when selectivity is governed by transport and partitioning; however, it depends on maintaining a thin or hierarchically porous architecture and ensuring adequate interfacial contact and electrical conductivity in electrocatalytic systems [183]. Otherwise, long diffusion paths and contact resistance can dominate, masking the intrinsic catalytic benefits.

Building on these cross-platform tendencies, polymer-interface selection should be guided by the primary operating constraints of the target system, including structural instability; microenvironment control (ion activity and local pH); mass transport and polarization in porous architectures; electronic wiring under high flux; and chemical robustness in harsh operating windows. Because these objectives are inherently coupled through the protection–accessibility and robustness–permeability balances, Table 2 summarizes the major trade-offs and design cues for each polymer class, providing an at-a-glance matrix that serves as a practical checklist for defining the initial interfacial design space.

## 4. Catalytic Application

### 4.1. Electrocatalysis

Electrocatalysis is an unusually revealing testbed for polymer-functionalized nanocatalysts because the measured performance is inseparable from interfacial ion transport, microenvironment, and electrode architecture [184,185]. Once a polymer is introduced, the interface is no longer a simple catalyst–electrolyte boundary: fixed charges, hydration state, and percolated ion pathways reshape how reactants and ions reach active sites, and they redefine the effective activities of protons, hydroxide, CO_2_, and spectator ions in the near-surface region [186,187]. As a result, polymer functionalization often shifts not only apparent activity but also selectivity and durability—especially at high current densities where concentration polarization and local pH gradients become dominant. Nevertheless, to elucidate the specific role of the polymer interphase, it remains important to clearly separate true intrinsic kinetic enhancement from apparent gains driven by changes in wetting, mass transport resistance, or catalyst-layer morphology; wherever possible, this distinction should be supported by kinetic normalization (e.g., electrochemical surface area (ECSA)-derived metrics), so that improvements attributed to polymer functionalization can be evaluated on an intrinsic basis rather than geometric area alone. Meaningful comparisons therefore require disentangling intrinsic catalytic changes from polymer-induced differences in wetting, transport resistance, and catalyst-layer morphology, and reporting activity/selectivity/stability under clearly specified cell configurations and operating regimes.

**Figure 10:**
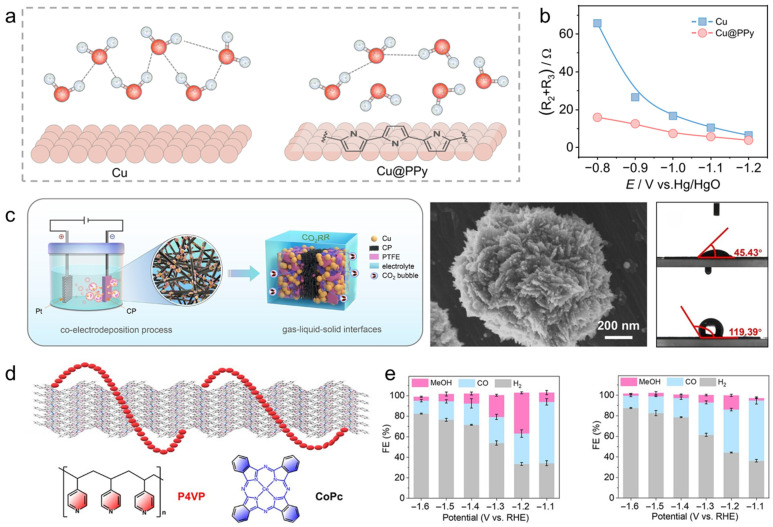
(**a**) A proposed scheme for interfacial water structure modulation over Cu and Cu@PPy. (**b**) Comparisons of the *E* a values of electrocatalytic deuteration of MCAA over Cu and Cu@PPy. Reprinted from Ref. [140] with permission from ACS. (**c**) Schematic illustration for the fabrication of Cu [CF_2_]_n_-x-CP and photographs of contact angle measurements. Reprinted from Ref. [189] with permission from Wiley-VCH. (**d**) Synthesis and characterization of core–shell Cu_2_O@COFs structures. A proposed molecular configuration of CoT-P. (**e**) Total FEs on CoT-P and CoT at various potentials. Reprinted from Ref. [191] with permission from Wiley-VCH.

Polymers can directly reconfigure interfacial electron distribution by introducing dipoles, redistributing the interfacial potential drop, and promoting charge delocalization across the catalyst–electrolyte boundary. These changes are mechanistically consequential because they shift adsorption energetics (particularly for polar intermediates), alter the driving force for elementary electron-transfer steps, and bias solvent orientation at the interface—effects that are amplified under operating polarization. A conductive PPy overlayer on Cu illustrates this “junction-enabled field” concept: the metal–polymer contact generates a built-in interfacial field that accelerates electron transfer and promotes favorable D_2_O reorientation, translating into a lower required overpotential and improved selectivity (Figure 10a,b) [140]. A related strategy uses a cationic conductive polymer interlayer (ABSA–PANI) between Cu nanoparticles and the electrolyte [188]. Rather than acting as a passive binder, the interlayer extends the electric double layer and increases the electron-transfer coefficient, while facilitating in situ generation of Cu–CO surface species; in this design logic, the polymer helps maintain a CO-rich reactive interface even under acidic conditions, yielding multicarbon selectivity above 81% at 600 mA cm^−2^ at pH 1.

Beyond electronic effects, polymers often operate as interfacial architects that simultaneously tune wetting, triple-phase boundaries, and local reactant activities. In CO2 electroreduction, the practical signatures of successful microenvironment design are familiar: suppressed HER, increased near-surface *CO coverage, and a higher probability of C–C coupling (or other selectivity-defining steps). One direct way to enforce this is to limit water/proton accessibility while maintaining CO_2_ supply (Figure 10c). Co-electrodeposition of PTFE with Cu on carbon paper creates a superhydrophobic gas–liquid–solid interface that biases the local balance toward CO_2_ availability and away from proton-rich pathways, enabling an ethylene FEs of 67.3% at −1.25 V vs. RHE (about 2.5× higher than Cu without PTFE) with stable operation for 11 h [189]. Notably, the polymer-modified electrode also exhibits a larger ECSA, as inferred from an increased double-layer capacitance, indicating improved interfacial accessibility. More importantly, the ECSA-normalized partial current density still increases, suggesting that the performance gain cannot be attributed solely to increased surface area but reflects an enhancement in site-level kinetics under the polymer-defined microenvironment. A complementary route uses polar/amine-rich polymers that enrich CO_2_ while stabilizing coupling intermediates. An amine-rich dipolar polymer (PAAz) couples electrostatic enrichment with hydrogen-bond stabilization of the *CO dimer, lowering the barrier for C–C dimerization while suppressing HER, and delivers an ethylene FEs of 68.9% at 1 A cm^−2^ [190]. More importantly, after ECSA normalization, Cu@PAAz still delivers the highest C_2_H_4_ partial current density, supporting that the activity enhancement is not solely a surface-area effect but is consistent with improved site-level kinetics under the polymer-defined microenvironment. In both cases, the polymer’s role is best framed as controlling local activities and residence times rather than merely increasing intrinsic activity.

Polymers can also behave as macromolecular ligand reservoirs, where pendant donor groups provide axial or secondary-sphere coordination to catalytic centers. This coordination can reorganize metal d-orbitals, shift the d-band center, and selectively stabilize rate- or branch-point intermediates (often *CO), enabling pathway steering without rebuilding the inorganic phase. A clear example is poly(4-vinylpyridine) (P4VP) coordinating Co centers in a cobalt phthalocyanine nanotube assembly (Figure 10d) [191]. The pyridyl coordination shifts the d-band center toward the Fermi level and strengthens *CO binding/activation—consistent with an increased population of CO antibonding character and a more activated C≡O geometry—leading to a ~14-fold increase in methanol-production turnover frequency and a methanol FEs of 40% at −1.2 V vs. RHE in 0.1 M KHCO_3_. In mechanistic terms, the polymer supplies a tunable coordination field that biases the surface toward the intermediate state required for productive branching. Across these electrocatalytic examples, polymers influence performance through three coupled levers—interfacial fields/electronics, microenvironment/transport, and coordination gating—so the central design task is not “add a polymer,” but co-optimizing polymer descriptors (coverage/thickness, charge/hydration, and ionic/electronic pathways) to ensure that selectivity gains are not offset by transport losses or unstable interfacial states.

### 4.2. Photocatalysis

In photocatalysis, polymer functionalization is compelling because it can tackle two bottlenecks with one interphase: (i) inefficient utilization of photogenerated carriers at the interface and (ii) poorly defined surface reaction environments [192,193]. Once a polymer is introduced, the interface becomes a programmable region that can (a) tune wettability and adsorption statistics, enrich reactants near photoactive sites, and build hydrogen-bonding or polar microdomains that stabilize short-lived intermediates; and (b) reshape charge separation and transfer by passivating surface traps, introducing interfacial dipoles, and acting as a molecular gate that regulates interfacial redox steps [194]. Accordingly, performance analysis should be framed around how the polymer couples photophysics (carrier generation, separation, lifetime) to surface chemistry (adsorption, intermediate stabilization, and desorption). A practical diagnostic is to ask whether the polymer’s primary contribution is improving carrier lifetime/transfer (electronic control) or redefining the near-surface microenvironment and transport (chemical control), while ensuring that light harvesting and active-site exposure are not compromised by excessive coverage or diffusion penalties.

Among polymer-enabled levers, heterojunction formation and built-in electric fields are especially direct routes to higher quantum efficiency. A heterojunction imposes an interfacial potential drop—via band bending, Schottky barriers, or S-/Z-scheme alignment—that spatially separates electrons and holes, suppressing recombination and biasing carriers toward the intended half-reactions. Polymer layers can amplify (or, if mis-designed, attenuate) this effect by providing oriented dipoles, modifying dielectric screening at the interface, and creating molecular-gating environments that favor directional carrier flow while maintaining permeability. The key design tension is therefore not “field strength” alone, but field strength under transport and exposure constraints: the same dipole-rich polymer that improves separation can also throttle mass transfer or shade active sites if the interphase becomes too thick, too dense, or poorly porous.

**Figure 11:**
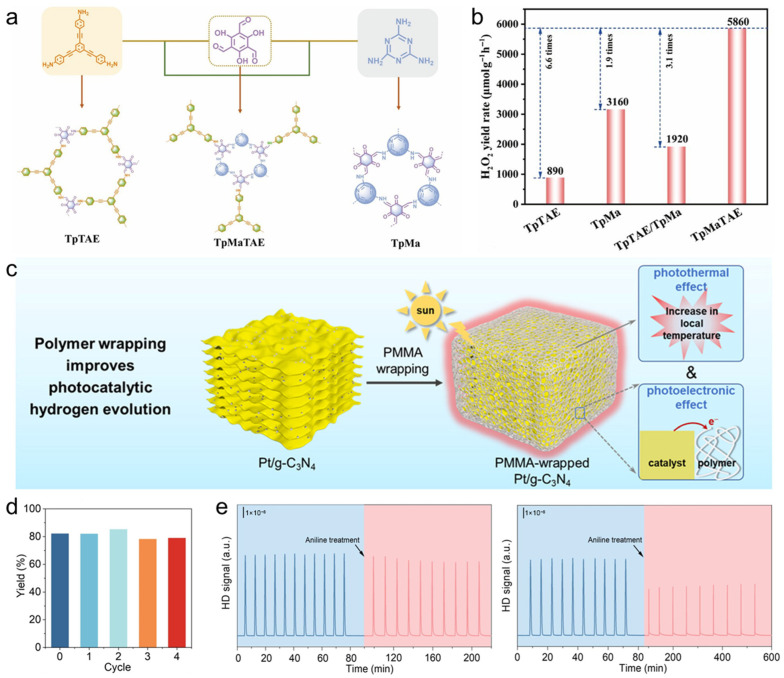
(**a**) Chemical structure of TpTAE, TpMa, and TpMaTAE. (**b**) Photocatalytic H_2_O_2_ generation of different photocatalysts in water. Reprinted from Ref. [195] with permission from Wiley-VCH. (**c**) The schematic diagram shows that polymer wrapping on the catalyst can enhance its photocatalytic hydrogen evolution capability. Reprinted from Ref. [196] with permission from Wiley-VCH. (**d**) Recycling tests of Pd/PDVB in the solvent-free hydrogenation of nitrobenzene. (**e**) H–D exchange experiments over Pd/PDVB and Pd/C with or without aniline adsorption. The blue and red colors indicate the results before and after aniline treatment, respectively. Reprinted from Ref. [197] with permission from ACS.

A dipole-driven built-in field can be achieved even within an all-organic photocatalyst. In a donor–acceptor–acceptor conjugated polymer (TpMaTAE), a large intramolecular dipole generates a strong internal electric field that reduces exciton binding energy and promotes unidirectional electron migration (Figure 11a) [195]. Carbonyl motifs further behave as redox-active electron reservoirs, extending carrier lifetime and enabling a relay-like electron supply to reduction sites; mechanistically, this is a “field + storage” coupling that both accelerates charge separation and increases the probability that electrons reach the reactive interface before recombination. Consistent with this picture, TpMaTAE delivers an H_2_O_2_ production rate of 5860 μmol g^−1^ h^−1^, together with 1.03% solar-to-chemical efficiency and 93.1% oxygen utilization using air and water (Figure 11b). Polymer overlayers can also reinforce charge separation in metal/semiconductor heterostructures while simultaneously tuning the local reaction environment. In a Pt/g-C_3_N_4_ system (where Pt acts as an electron sink that establishes an interfacial field), wrapping with PMMA provides a transparent, conformal interphase that is argued to preserve micro-/submicron pores for transport, introduce additional surface fields via polymer dipoles, and retain photothermal energy because PMMA is thermally insulating (Figure 11c) [196]. This combination is mechanistically attractive because it couples faster carrier extraction with locally accelerated surface kinetics without forcing a trade-off between electronic gains and interfacial accessibility. Experimentally, PMMA wrapping yields an ~1.5× higher HER rate, increases AQY at 420 nm from 3.44% to 7.62%, and reduces illuminated interfacial charge-transfer resistance to 134.9 Ω, consistent with improved separation and transfer alongside maintained transport. Across polymer-functionalized photocatalysts, the most transferable design rule is to treat polymers as coupling layers rather than coatings: the target is an interphase that strengthens charge separation/transfer and stabilizes the desired surface pathway while remaining optically transparent, permeable, and thin enough to keep active sites accessible.

### 4.3. Thermocatalysis

Thermocatalysis is a key arena where polymer functionalization can deliver practical value through structural stabilization, selective permeability, and microenvironment control, particularly for nanocatalysts that suffer from sintering, coking, or leaching under elevated temperature and reactive atmospheres [198,199]. Relative to fully inorganic shells, polymer-derived or hybrid interphases offer a rare combination of conformal contact and tunable permeability, enabling protection without fully shutting down reactant access to the active surface. Polymer chemistry also provides an extra lever in liquid-phase thermocatalysis, where selectivity is often dictated by interfacial polarity and solvation-like effects rather than by the metal identity alone. The core challenge is therefore an engineering trade-off: stabilize the catalyst while avoiding transport throttling, and ensure the interphase itself remains chemically and thermally intact [200]. For this reason, claims of performance improvement are most convincing when supported by time-on-stream stability, rigorously normalized rates, and mechanistic deactivation analysis (sintering, coke buildup, phase transformation).

A recurring mechanistic theme in polymer-enabled (and polymer-like porous) thermocatalysis is that selectivity can be rewritten by controlling adsorption geometry and interfacial hydrogen availability, even when the metal phase is unchanged. One instructive framework example embeds Pt nanoparticles within a Ni-node environment that preferentially engages the substrate C=O functionality, effectively “pre-organizing” adsorption so that the competing bond is presented to Pt for hydrogenation [201]. This geometric steering becomes more powerful when paired with hydrogen management: low H_2_ spillover helps localize reactive hydrogen near productive ensembles, sharpening chemoselectivity, while partial in situ reduction in Ni nodes can introduce Ni nanoparticles that accelerate H_2_ activation without erasing the adsorption-directing role of the surrounding framework. The broader implication is that the organic/porous microenvironment can decouple where H_2_ is activated from how the substrate is oriented at the active metal surface.

A second thermocatalysis-relevant role is anti-poisoning through product management, which becomes decisive under solvent-free or high-concentration conditions where desorption is rate-limiting and product inhibition dominates. In Pd-catalyzed nitrobenzene hydrogenation, porous poly(divinylbenzene) (PDVB) provides an aromatic scaffold that preferentially captures aniline through π-π interactions, reducing the probability that aniline saturates Pd and suppresses H_2_ activation (Figure 11d,e) [197]. In this picture, the polymer is not merely a container; it acts as a product sink that continuously drains inhibiting species from the active surface, thereby sustaining turnover and simultaneously suppressing leaching. Consistent with this mechanism, kinetic and isotopic probes (including H-D exchange) support the view that aniline adsorption strongly hinders H_2_ dissociation on conventional supports, whereas the PDVB microenvironment mitigates that penalty by redistributing aniline away from Pd.

Beyond confinement and partitioning, functional-group electronics provide a molecular lever to tune hydrogen activation energetics by reshaping local acid-base character. In a functionalized MOF-808 series (MOF-808-X), substituents spanning electron-donating to electron-withdrawing character systematically modulate the strength and population of basic sites, evidenced by shifts in CO_2_ desorption features and probe-IR signatures. Mechanistically, electron-donating groups can transmit electron density through the ligand backbone to reinforce the Lewis-base component of adjacent frustrated-Lewis-pair-like sites, thereby lowering the barrier for heterolytic H_2_ cleavage through a push–pull pathway [202]. Consistent with this rationale, the amine-functionalized variant exhibits markedly stronger hydrogenation performance (e.g., complete conversion under the reported conditions) than the parent material or electron-withdrawing analogs, underscoring that organic microenvironments can tune the thermodynamics of H_2_ activation without altering the metal phase itself.

Taken together, these cases point to three transferable design handles for polymer-enabled thermocatalysis: (i) adsorption pre-organization that biases bond presentation, (ii) product partitioning that suppresses poisoning in desorption-limited regimes, and (iii) functional-group electronics that tune heterolytic H_2_ activation. The most durable designs emerge when these chemical effects are matched to a permeability architecture that preserves flux while resisting sintering, coking, and leaching.

## 5. Industrial Translation and Real-World Adoption Considerations

Despite the conceptual maturity of polymer-enabled interfacial design, translating polymer-functionalized nanocatalysts into industrially relevant systems remains challenging [25,203,204,205,206]. Laboratory performance is often inseparable from electrode architecture, operating flux, and long-term chemical and mechanical stability. In practice, a polymer layer that improves selectivity or apparent activity at small scale can behave differently in thick, porous catalyst layers, where wetting, capillary pressure, gas–liquid management, and ionic and electronic percolation jointly determine catalyst utilization and polarization losses [207]. Therefore, industrial translation requires treating polymers not only as molecular modifiers but also as manufacturable interphase materials, whose thickness uniformity, continuity, and defect tolerance must be controlled over large areas while maintaining low transport resistance and stable triple-phase boundaries under dynamic operating conditions.

Cost and scale-up impose additional constraints that are often underemphasized in proof-of-concept studies. Some advanced platforms (e.g., COF- or MOF-derived shells and designer conductive polymers) require multistep synthesis, solvent-intensive processing, and expensive monomers or linkers, and may need post-treatments or activation steps that reduce throughput and reproducibility [208]. Even for scalable binders and ionomers, cost is not limited to the polymer itself, because processing overhead—such as ink formulation windows, coating rheology, drying/annealing energy, solvent recovery, and roll-to-roll compatibility—can dominate [209,210]. Durability at scale is equally important: polymer interphases must resist swelling-induced cracking, delamination, redox-driven chemical attack, and contamination or fouling over thousands of hours, while maintaining stable transport pathways and interfacial microenvironments across variable feed compositions.

To accelerate real-world adoption, future work would benefit from tighter alignment between polymer/interphase metrics and device-level figures of merit. This includes reporting polymer descriptors relevant to manufacturability (e.g., solids content, viscosity/rheology, adhesion/cohesion, and water uptake/swelling), quantifying stability under relevant accelerated stress tests, and benchmarking performance in standardized configurations (e.g., membrane–electrode assemblies, flow cells, and slurry or packed-bed reactors) rather than only in idealized half-cells. To improve cross-study comparability and enable transferable design rules, we recommend a minimum reporting set for polymer-functionalized catalysts, where feasible, that covers both interphase descriptors and device context. At a minimum, future studies should report the following:

(1) Interphase geometry: polymer thickness (or effective layer thickness) and coverage/continuity, together with the deposition/anchoring route;
(2) Charge and hydration: fixed-charge metrics (e.g., charge density or IEC, or experimentally accessible proxies) and swelling/water uptake under relevant conditions;
(3) Transport properties: ionic conductivity and/or electronic conductivity (as applicable), and at least one indicator of permeability/porosity relevant to reactant/product flux;
(4) Electrode architecture and utilization: catalyst loading, catalyst-layer thickness/porosity (or a comparable structural descriptor), wetting/contact-angle trends, and an accessibility proxy such as ECSA/C_dl_ when relevant;
(5) Testing transparency and normalization: cell configuration and operating regime (electrolyte composition, flow/pressure where applicable), and normalization choices (e.g., ECSA-normalized partial current densities) to distinguish intrinsic kinetic changes from transport- or morphology-driven artifacts.

Integrating techno-economic and life-cycle considerations—such as polymer availability, recyclability, and end-of-life handling—can further clarify which polymer platforms are most promising for scale-up. Overall, industrial translation will likely rely on polymer strategies that deliver microenvironment control and stability without adding large-area coating complexity or transport penalties, as well as design rules that remain robust across processing and operating windows.

## 6. Conclusions

Polymer functionalization has emerged as a broadly applicable and conceptually powerful strategy to upgrade nanocatalysts, not by altering the catalytic phase alone, but by programming the interface where catalysis occurs. Across diverse catalytic platforms, polymers offer an unusual combination of design freedom and processability, enabling conformal and tunable interphases that mitigate persistent limitations of nanocatalysts, including aggregation and reconstruction, unstable coordination environments, poorly controlled local reaction conditions, and transport bottlenecks. Rather than acting as passive binders, polymers function as active interfacial materials that couple structural stabilization with microenvironment regulation and flux management, thereby transforming high-surface-area catalysts into functionally engineered catalytic interfaces.

Organizing the field around functional polymer platforms highlights several general lessons. Neutral organic polymers effectively stabilize dispersed nanophases and tune wettability with minimal architectural complexity, whereas ionomers and polyelectrolytes excel at reshaping local ionic environments and enabling ion conduction in porous electrodes, thereby often dictating selectivity under high-rate operation. Conductive polymers uniquely integrate interfacial chemistry with charge transport, while crosslinked networks and hydrogels create confined microreactors that combine durability with controlled diffusion. Hybrid inorganic–organic polymers provide robust protective interphases under harsh conditions, whereas framework-type polymeric materials introduce topology-defined porosity that enables molecular sieving, site isolation, and reactant enrichment as an additional layer of selectivity control. Across these classes, catalytic outcomes are governed by a coupled set of polymer parameters—thickness and coverage, charge density, swelling and permeability, and ionic/electronic conductivity—that must be co-optimized to balance protection, accessibility, and transport.

Design principles (take-home messages): (i) Select the platform based on the dominant bottleneck (structural instability, microenvironment control, transport/polarization, electronic wiring, or robustness under harsh operating windows), rather than polymer identity alone. (ii) Treat thickness/coverage and permeability as first-order design variables, because overly thick or continuous coatings can turn stabilization into site blocking or diffusion limitation. (iii) Co-design microenvironment regulation with flux management: parameters that strengthen local control (e.g., charge density and hydration in ionomers; crosslinking and swelling in gels) should be tuned to avoid transport penalties. (iv) Engineer percolation explicitly for high-flux operation, where ionic/electronic connectivity and interfacial contact can dominate the apparent kinetics. (v) Aim for selectively protective interphases in which durability gains come from suppressing corrosion and fouling while maintaining access and minimizing interfacial resistance. (vi) Support causal attribution with normalization and transparent reporting, and distinguish intrinsic kinetic enhancement from morphology- or transport-driven artifacts whenever possible.

Looking ahead, the most impactful advances will likely come from moving beyond empirical coating strategies to quantitative, transferable design rules. Achieving this will require more consistent reporting of polymer characteristics and electrode architecture, clearer separation of intrinsic catalytic effects from transport- or morphology-driven artifacts, and stronger links between polymer structure, interfacial microenvironments, and reaction pathways. In parallel, polymer-defined interfaces are well-positioned to unlock underexplored applications in which microenvironment and transport constraints are especially decisive. Examples include nitrogen-cycle electrocatalysis (e.g., nitrogen conversion and nitrate/NO_x_ reduction), aqueous-phase biomass upgrading (where phase behavior and substrate partitioning often limit performance), and tandem or cascade catalysis (where compartmentalization and intermediate relay distances can determine efficiency and selectivity). Future strategies are also likely to leverage dynamic and “smart” polymer interphases that respond to operating conditions; for example, they may tune permeability, hydration state, or charge density in situ to stabilize local reactant activities and suppress degradation pathways. Related opportunities include self-healing or reconfigurable coatings that restore interfacial continuity after swelling-induced cracking, delamination, or mechanical stress, thereby extending lifetime under device-relevant flux and cycling. Finally, as descriptor reporting becomes more standardized, data-driven and machine-learning-guided approaches may offer a practical route to navigating the large polymer design space and identifying architectures that achieve targeted microenvironments with minimal transport penalties. Together with framework and hybrid architectures that integrate microenvironment engineering with long-term stability, these directions reinforce the view of polymers as programmable interfacial materials and position polymer functionalization as a central strategy for delivering simultaneous gains in activity, selectivity, and durability in next-generation nanocatalyst systems.

## Figures and Tables

**Table 1:** Descriptor–mechanism–performance map and cross-class comparison of polymer interfaces in catalysis.

Polymer Type	Stabilization Capability	Microenvironment Regulation	Transport Management	Mechanistic Implications for Catalysis	Scalability	Typical Drawbacks
Neutral functional polymers	Improve adhesion and provide partial shielding; effectiveness depends on conformal yet permeable coverage.	Tune local polarity/solvation and interfacial dielectric environment near active sites.	Often adds diffusion resistance unless permeability/porosity is engineered.	Durability often improves; activity may increase or decrease depending on access vs blocking; selectivity shifts are system-dependent.	High; simple coating/binder-type processing.	Protection–accessibility balance; thickness/coverage can penalize mass transport.
Ionomers/polyelectrolytes	Anchor particles and help stabilize interfacial composition; sensitive to mechanical integrity.	Fixed charges regulate ion partitioning and local ion activity (including local pH).	Strong ionic pathways when hydrated; electronic transport often requires hybridization.	Can improve high-rate activity (reduced ionic polarization) and bias selectivity via ion/pH microenvironment; durability depends on swelling control.	High; widely compatible with electrode fabrication.	Hydration benefits transport but risks swelling/flooding; excessive thickness can impose diffusion losses.
Conductive polymers	Provide binding and partial protection; durability limited by oxidative/redox stability.	Modulate local fields/wettability and specific interactions; effects depend on doping state.	Strong electronic wiring; ionic transport varies with morphology and hydration.	Activity may improve via better charge delivery; selectivity modulation is possible but non-universal; aging depends on chemical/electrical continuity.	Moderate; processable but stability/formulation constraints.	Conductivity–permeability–stability coupling; dopant migration/redox cycling can cause drift.
Crosslinked networks/hydrogels	Robust encapsulation can suppress detachment/aggregation when crosslinking is optimized.	Confinement and functional groups regulate local water/ion structure (mesh-size-dependent).	Permeability set by mesh and swelling; electronic transport typically limited unless hybridized.	Durability often improves; activity/selectivity reflect confinement benefits vs diffusion limitations.	Moderate; crosslinking steps add complexity.	Swelling–mechanics trade-off; overly dense networks lead to concentration polarization.
Hybrid inorganic–organic polymer layers	Strong barrier effect and chemical robustness under harsh conditions.	Moderate regulation via polarity gradients, acid–base sites, and water management.	Variable; limited by interfacial resistance and connectivity/free volume.	Durability often increases; activity/selectivity depend on maintaining access and minimizing resistive interfaces.	Moderate; integration can be more involved.	Barrier strength and accessibility; brittleness/contact resistance may rise with inorganic fraction.
Framework-type porous polymers	Stabilize sites via rigid scaffolding if framework is stable in the environment.	Defined pores enable sieving, tailored adsorption fields, and confinement-driven microenvironments.	Variable; performance hinges on conductivity and contact engineering.	Selectivity often reflects size/interaction discrimination; activity depends on transport/contact; durability constrained by stability window.	Low to moderate; film/contact engineering can be demanding.	Stability window; integration complexity; thick films increase resistance and diffusion length.

**Table 2:** Cross-platform trade-off matrix for polymer-functionalized catalyst interfaces.

Polymer Type	Primary Strength	Dominant Trade-Offs to Manage	Practical Design Cue
Neutral functional polymers	Soft, conformal stabilization with broad compatibility	Coverage/thickness that stabilizes can also mask sites and add diffusion resistance	Prefer thin/permeable layers; tune anchoring density to avoid “over-coating”
Ionomers/polyelectrolytes	Program ion activities and local pH; enable ionic pathways in porous layers	Higher charge density/hydration improves microenvironment control but can increase swelling, flooding, and transport polarization	Co-optimize charge density, water uptake, and pore connectivity; avoid continuous thick films
Conductive polymers	Electronic percolation plus interfacial chemistry control	Higher conductivity (doping) can couple to redox/oxidative instability; thick skins raise mass-transfer losses	Use thin, porous, robustly anchored networks; prioritize stability of doping state under operation
Crosslinked networks/hydrogels	Microreactor confinement and deactivation/intermediate management	Mechanical robustness (high crosslinking) reduces permeability; high swelling improves flux but can weaken integrity	Engineer hierarchical porosity/channels; match mesh size to diffusion length scales
Hybrid inorganic–organic polymer layers	Harsh-window robustness; selective barrier behavior	Stronger protection often increases interfacial resistance and access penalties; high inorganic content can raise brittleness	Keep coatings thin and defect-tolerant; design selective permeability rather than dense blocking layers
Framework-type porous polymers	Pore-defined enrichment/exclusion; site isolation	Greater thickness/order improves sieving but increases diffusion length/contact resistance; stability window constraints	Use thin/hierarchical shells; ensure electrical/ionic contact and chemical stability in the target medium

## Data Availability

No new data were created or analyzed in this study. Data sharing is not applicable to this article.
